# *Xenopus* as a Model System for the Study of GOLPH2/GP73 Function: *Xenopus* golph2 Is Required for Pronephros Development

**DOI:** 10.1371/journal.pone.0038939

**Published:** 2012-06-14

**Authors:** Leike Li, Luan Wen, Yu Gong, Guoqiang Mei, Jinsong Liu, Yonglong Chen, Tao Peng

**Affiliations:** 1 State Key Laboratory of Respiratory Disease, Guangzhou Institute of Biomedicine and Health, Chinese Academy of Sciences, Guangzhou, China; 2 University of Science and Technology of China, Hefei, China; 3 Key Laboratory of Regenerative Biology, Guangzhou Institute of Biomedicine and Health, Chinese Academy of Sciences, Guangzhou, China; 4 Section on Molecular Morphogenesis, Laboratory of Gene Regulation and Development, Program in Cellular Regulation and Metabolism, Eunice Kennedy Shriver National Institute Child Health and Human Development, National Institutes of Health, Bethesda, Maryland, United States of America; Institute of Molecular and Cell Biology, Singapore

## Abstract

GOLPH2 is a highly conserved protein. It is upregulated in a number of tumors and is being considered as an emerging biomarker for related diseases. However, the function of GOLPH2 remains unknown. The *Xenopus* model is used to study the function of human proteins. We describe the isolation and characterization of *Xenopus* golph2, which dimerizes and localizes to the Golgi in a manner similar to human GOLPH2. *Xenopus golph2* is expressed in the pronephros during early development. The morpholino-mediated knockdown of golph2 results in edema formation. Additionally, *Nephrin* expression is enhanced in the glomus, and the expression of pronephric marker genes, such as *atp1b1, ClC-K, NKCC2,* and *NBC1*, is diminished in the tubules and duct. Expression patterns of the transcription factors *WT1, Pax2, Pax8, Lim1, GATA3,* and *HNF1β* are also examined in the golph2 knockdown embryos, the expression of *WT1* is increased in the glomus and expanded laterally in the pronephric region. We conclude that the deletion of golph2 causes an increase in the expression of *WT1*, which may promote glomus formation and inhibit pronephric tubule differentiation.

## Introduction

Human Golgi phosphoprotein 2 (GOLPH2, also termed GP73 and GOLM1) is a type II transmembrane protein that resides in the *cis*- and *medial*-Golgi cisternae and is primarily expressed in the epithelial cells of several different human tissues [1]. The upregulation of GOLPH2 expression has been correlated with many diseases and viral infections, including liver disease, prostate cancer, and renal cell cancer [2], [3], [4], [5], [6].

Despite its association with various diseases, little is known regarding the function of GOLPH2. Based on protein sequence analyses, GOLPH2 is conserved among different species, with all family members containing a short cytoplasmic N-terminus, a transmembrane domain (TMD) and a coiled-coil domain. Previous studies indicated that GOLPH2 has the following characteristics: first, human GOLPH2 exists as a dimer stabilized by two inter-protein disulfide bonds formed by two conserved cysteine residues in the coiled-coil domain [7]; second, Golgi localization of GOLPH2 is determined by the TMD and a single positively charged residue extending from the TMD on the cytoplasmic side; and third, human GOLPH2 is mainly expressed in epithelial cells [1].

To determine the possible cellular functions of GOLPH2, a transgenic mouse model that expressed a mouse GOLPH2 C-terminal truncation was generated [8]. This mouse displayed decreased survival and severe epithelial abnormalities in the kidneys, thereby suggesting that mouse GOLPH2 might play an important role in the formation and function of this organ.

The vertebrate kidney is a complex organ that maintains water and salt homeostasis and excretes waste products [9]. Three distinct kidneys develop in succession: the pronephros, mesonephros and metanephros. The pronephros is the functional embryonic kidney of amphibians. It is composed of a single nephron that contains the blood-filtering glomus, the proximal-distal tubules and the pronephric duct [10]. Each segment of the pronephros contains distinct domains, as evidenced by the differential expression of several membrane transporter genes. The pronephros is structurally and functionally similar to the nephron in the metanephric kidney [11], [12]. Studies in *Xenopus* and mice have suggested that Notch signaling pathways [13], [14], as well as the transcription factors *Lim1, Pax2/Pax8, WT1, HNF1β* and *GATA3*, are involved in the formation of the pronephros [15]. Most of the important regulatory molecular components that express during early embryonic development are conserved in vertebrates [16]. Due to its relatively simple organization and due to the ease with which it can be genetically manipulated, *Xenopus* has been used as a model system to study the biochemical pathways and conserved protein functions involved in the complex regulation of organogenesis, including early kidney development [17], [18].

The predicted amino acid sequences of *Xenopus* golph2 showed 57% similarity with human GOLPH2. The most conserved regions are the ones that contain the cytoplasmic tail, the TMD and the coiled-coil domain at the N-terminus of the protein (Fig. S1). Based on this observation, we hypothesized that protein function is conserved between golph2 and GOLPH2; therefore, with the goal of understanding the biological function of GOLPH2 and taking advantage of the *Xenopus* model system, we sought to determine whether we could observe any phenotypic changes following inhibition of golph2 expression during *Xenopus* development.

Here, using *Xenopus* as a model, we demonstrated that golph2 exhibits properties similar to those of GOLPH2, such as intracellular localization, epithelial specific cellular expression, and dimer formation, thereby indicating that the two homologs might also perform similar biological functions. Following the observation of the pronephric expression profiles of *golph2* during early embryonic development, we analyzed the function of golph2 and its requirement for pronephros development. We demonstrated that the inhibition of golph2 translation expands the expression of a glomus marker and reduces the expression of pronephric markers in the tubules and duct. These results suggest that golph2 is required for the terminal differentiation of the pronephros.

## Materials and Methods

### Ethics Statement

This study was carried out in strict accordance with the recommendations in the Guide for the Care and Use of Laboratory Animals of the National Institutes of Health. The protocol (2011035) was approved by the IACUC committee of the Guangzhou Institute of Biomedicine and Health, Chinese Academy of Sciences.

### Plasmids and RT-PCR

The complete open reading frame with 5′ UTR of *golph2* (GenBank accession number JF79249) was amplified from the cDNA of stage 40 *Xenopus laevis* embryos and cloned into the pCS2+ vector (pCS2+-*golph2*). The following primers were used: forward, CTGCGGATCCACGGAAACAAGCTGTGTGGCCTTA; reverse, TGGCAGAACGTGCTCTAGATTATTTCTTAAGGAGTGGAATATGCTC. Mutations within the morpholino binding site were introduced by PCR-based site-directed mutagenesis into the vector pCS2+-*golph2.* The mutated plasmid was constructed for generation of *golph2 rescue* mRNA. The following primers were used: forward, CAAGGTTTGCACCTGATTGTGAGATGATGGGCTCGGGTAATGGTC, reverse, GACCATTACCCGAGCCCATCATCTCACAATCAGGTGCAAACCTTG. The ORF of human *GOLPH2* was cloned into either the pCS2+ or pCR3.1 vector (containing a FLAG tag).

The probe for whole-mount *in situ* hybridization reactions was generated by amplifying a partial sequence of *golph2* using the following PCR primers: forward, GCTGGTCGACAAAAGACTCAGAAACCCAAGA; reverse, GATTACGCATGCACTATACCCAGCAGAACAGAT. The resulting fragment was subcloned into the pGEM-T Easy vector (Promega, USA). All pronephric marker genes used in this study were also cloned into this vector (The primer sequences are listed in Table S1).

*Xenopus* golph2Δ1–34 (deletion of the first 34 N-terminal residues that include the TMD and cytoplasmic tail) was cloned into the pET22b (+) vector for expression.

Semi-quantitative RT-PCR was performed using the following primers: *golph2*: forward, TAAGAAATAAAGGCACGCACAT, reverse, CTCACTATACCCAGCAGAACAGA; *ornithine decarboxylase* (*ODC*): forward, GGAGCTGCAAGTTGGAGA, reverse, TCAGTTGCCAGTGTGGTC.

The stalk region of Golgi protein galactosyl-*N*-acetyltransferase (GalNAcT2) was fused to the N-terminus of EGFP [19]. A 342 bp fragment (amino acids, 1–114) from human hGalNAcT2 cDNA (NCBI accession number X85019), and a 310 bp fragment (amino acids, 1–103) from *Xenopus* xGalNAcT2 cDNA (NCBI accession number JX014219) were generated by PCR. The following primers were used: hGalNAcT2: forward, ATCCGCTCGAGATGCGGCGGCGCTCGCGGATG, reverse, GGATGGGAATTCCCATTCGAAGCTTATCACTCTCCAC; xGalNAcT2: forward, TATCGCAGATCTGGTCAGGGAGCCCAGGAGAGAAACAATG, reverse, CGTGTCAGGAACACGAATTCCCATCAGGAGTTTATCACTTTCAACT. The resulting fragments were cloned into pEGFP-N1 vector. Then the fusion fragments containing stalk region and EGFP were subcloned into pCS2+ vector.

### Protein Expression and Purification

The golph2Δ1–34 fusion protein was expressed in *Escherichia coli* strain BL21 (DE3). The soluble protein was purified by Ni-NTA affinity chromatography (Qiagen, USA) and ion-exchange chromatography (SOURCE 15Q, GE Healthcare, USA). The purified protein was used as an antigen to produce a mouse monoclonal antibody (10F12), which was used for Western blotting, immunoprecipitation and immunofluoresence analysis of golph2 in this study. The antibody for *Xenopus* golph2 does not cross-react with human GOLPH2.

To detect endogenous golph2 proteins, embryos at different stages were homogenized in RIPA buffer (50 mM Tris, pH 7.4; 1% Triton X-100; 1% sodium deoxycholate; 0.1% SDS; 2 mM EDTA; 1 mM Na_3_VO_4_; 100 mM NaF; 1 mM PMSF; and phosphatase inhibitor cocktail). The lysates were centrifuged at 15,000×g for 20 min, 4°C and the supernatant was heated in loading buffer containing 140 mM 2-mercaptoethanol. One embryo per lane was separated by SDS-PAGE and detected by Western blotting with anti-golph2 antibody 10F12, human GOLPH2 was detected with antibody 5B12 (from our lab) [20]. For nonreducing SDS-PAGE, samples were heated in loading buffer without 2-mercaptoethanol.

### Immunoprecipitation, Immunofluorescence and Immunocytochemistry

The immunoprecipitation experiment was performed as previously described [7]. Briefly, both pCS2+-*golph2* and pCR3.1-*GOLPH2*-FLAG were co-transfected and expressed in 293 T cell lines. Total cell lysates were prepared at 36 hours post-transfection. Purified mouse anti-golph2 antibody 10F12 and protein G Plus/Protein A agarose (Calbiochem, USA) were used to immunoprecipitate golph2 from the lysates. Immunoprecipitates were resolved by SDS-PAGE and immunoblotted with anti-FLAG antibody M2 (Sigma, USA) to detect human GOLPH2.

Stage 46/47 embryos were fixed in MEMFA (0.1 M MOPS, pH 7.4; 2 mM EGTA; 1 mM MgSO_4_ and 3.7% formaldehyde) for one hour at room temperature, embedded in paraffin, and sectioned at a thickness of 3 µm. The sections were boiled in 1 mM EDTA (pH 8.0) to unmask antigenic sites. Endogenous golph2 was localized using the antibody 10F12 followed by incubation with a Cy3-conjugated secondary antibody (Chemicon, USA) or HRP-conjugated secondary antibody (Zhongshan Golden Bridge, China).

For the animal cap assay, the mRNA of hGalNAcT2-EGFP or xGalNAcT2-EGFP was injected into the animal pole of the fertilized eggs. Animal caps were dissected from stage 9 embryos, cultured in 0.8×MBS, and fixed at stage 21 with Dent’s Fixative overnight at -20°C. Animal caps were permeabilized with PBS containing 0.3% Triton X-100 for 30 min at room temperature. Endogenous golph2 was stained with the antibody 10F12 followed by incubation with a Cy3-conjugated secondary antibody (Chemicon, USA). Nuclei were counterstained with 4, 6-diamidino-2-phenylindole (DAPI; KPL, USA). Both the sections and cells were visualized using a confocal microscope (LSM 710; Zeiss, Germany).

### Embryo Manipulations, Synthetic MRNA, and in Situ Hybridization

*Xenopus laevis* embryos were obtained by *in vitro* fertilization as previously described [21] and staged according to Nieuwkoop and Faber [22]. Capped mRNAs were synthesized *in vitro* from NotI linearized plasmids using the mMESSAGE Machine SP6 Kit (Ambion, USA). The following plasmids were used: pCS2+-*golph2*, pCS2+-*golph2*-*allele*, pCS2+-*golph2*-*rescue*, pCS2+-*GOLPH2,* pCS2+*-hGalNAcT2-EGFP* and pCS2+*-xGalNAcT2-EGFP*. To generate digoxigenin-labeled antisense probes, pGEM-T Easy-*golph2* was linearized with SalI and transcribed with T7 RNA polymerase (Promega, USA). The following probes for marker genes were cloned into the pGEM-T Easy vector: *Nephrin* [23], *atp1b1* [18], *SGLT-1K* [11], *ClC-K* [24], *NKCC2* [11], *NBC1* [11], *WT1* [25], *Lim1* [26], *Pax2* [26], *Pax8* [26], *HNF1β* [27], and *GATA3* [18]. Whole-mount *in situ* hybridization was performed as previously described [28]. The images were taken using a stereomicroscope (SZX16; Olympus, Japan) equipped with a CCD camera (DP72; Olympus, Japan). Gelatin/albumin sections (30 µm) were prepared using a vibratome as previously described (VT1000S; Leica, Germany) [29].

### Morpholino Oligonucleotides and Microinjection

Morpholino against *golph2*, 5′-CCTGATCCCATCATTCTGCAGCAGC-3′, was designed to target the translation start site, *golph2*-MO and the standard Control-MO oligos, 5′-CCTCTTACCTCAGTTACAATTTATA-3′, were suspended in sterile water to a concentration of 1 mM (Gene Tools, USA). For the marker genes analysis, 32 ng of *golph2*-MO was injected into the marginal zone of one ventral blastomere at the 4-cell stage along with 10 KD fluorescein-dextran (Molecular Probes, USA) as a lineage tracer. The embryos were cultured and fixed at various stages.

## Results

### 1. Similar to GOLPH2, *Xenopus* Golph2 is a Golgi-localized Dimer and Expressed in Epithelial Cells

**Figure 1:**
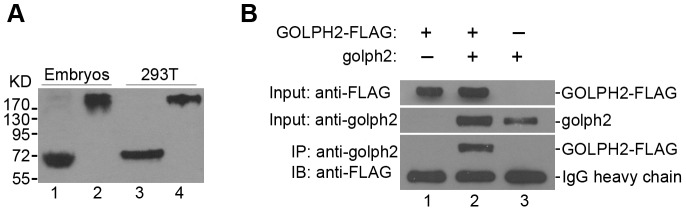
*Xenopus* golph2 forms a disulfide bond-linked dimer and interacts with human GOLPH2. A. Whole lysates were prepared from stage 22 embryos of *Xenopus* and 293 T cell lines; the supernatant was boiled with loading buffer with (lines 1 and 3) or without (lines 2 and 4) 140 mM 2-mercaptoethanol. Samples were resolved on a 12% SDS-PAGE gel and analyzed by Western blotting with anti-golph2, 10F12, (lines 1 and 2) and anti-GOLPH2, 5B12 (lanes 3 and 4). **B**. The 293 T cell line was co-transfected with pCS2+-*golph2* and pCR3.1-*GOLPH2*-FLAG (line 2) or a single construct with the empty vectors (lines 1 and 3). Cell lysates were prepared at 36 hours post-transfection (Input). The supernatant was immunoprecipitated (IP) with an anti-golph2 antibody, 10F12, and the captured proteins were immunoblotted (IB) with an anti-FLAG antibody.

It has been shown that human GOLPH2 is a dimer [7]. To determine whether golph2 also exists as a disulfide bond-linked dimer, endogenous golph2 from stage 22 embryos was analyzed using nonreducing SDS-PAGE and detected by Western blotting. As shown in Fig. 1A, golph2 has the same migration pattern as the human GOLPH2 protein. Furthermore, the Golgi segment of *Xenopus* golph2 was expressed and purified (from 117 to the C terminal of the protein). The molecular mass is around 70 KD, determined by size fractionation column (data not shown). The theoretical molecular mass for monomer is 32.4 KD. It suggests that golph2 is a dimer. Because the dimerization of GOLPH2 is mediated via an interaction between the conserved coiled-coil domains, we sought to determine whether golph2 could interact with GOLPH2 when the two proteins are co-expressed. The plasmids expressing GOLPH2-FLAG and golph2 were transiently transfected in 293 T cells. As shown in Fig. 1B, GOLPH2-FLAG co-precipitated with golph2 (compare lane 2 with lanes 1 and 3), thus indicating that golph2 is capable of forming a hetero-complex with GOLPH2.

**Figure 2:**
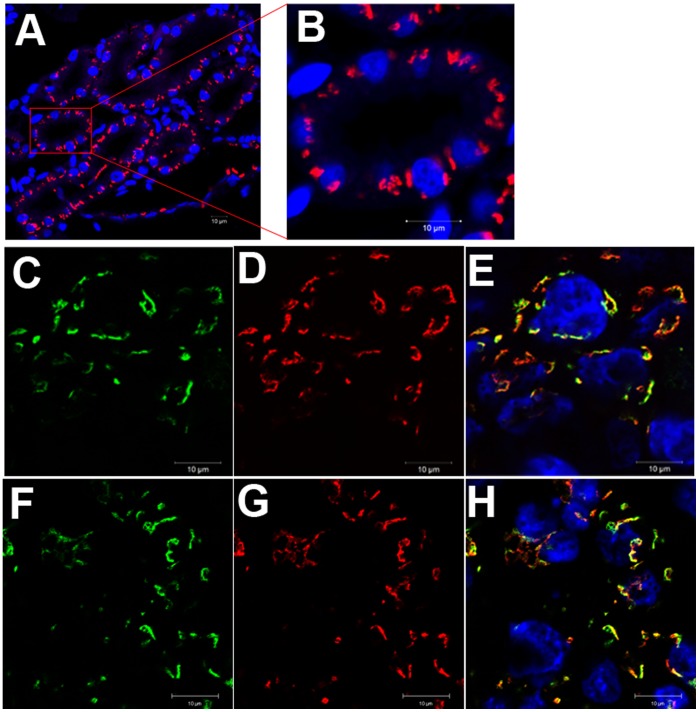
*Xenopus* golph2 localizes to the Golgi. A. Perinuclear localization of endogenous golph2 in the pronephros of stage 46/47 embryos. **B.** Pronephric tubule. **C–E.** Endogenous golph2 co-localizes with hGalNAcT2-EGFP in animal cap cells. The mRNA of hGalNAcT2-EGFP was injected into the animal pole of the fertilized eggs (C, green); golph2 was detected using the antibody 10F12 followed by Cy3-conjugated secondary antibody (D, red); merge (E). **F–H.** Endogenous golph2 co-localizes with xGalNAcT2-EGFP in animal cap cells. xGalNAcT2-EGFP (F, green); golph2 (G, red); merge (H). Bar = 10 µm.

Previously, we demonstrated that the Golgi retention of GOLPH2 is determined by the TMD [7]. The TMD of golph2 is highly conserved with GOLPH2, thereby suggesting that golph2 may localize to the Golgi. As shown in Fig. 2A, perinuclear localization of endogenous golph2 was observed in stage 46/47 embryonic pronephros. It has been shown that N-terminal of human hGalNACT2 localized in the Golgi of *Xenopus* A6 cell lines [19]. We use the EGPF-fusion protein of hGalNACT2 and the *Xenopus* xGalNACT2 as Golgi markers. We detected that endogenous golph2 co-localized with both of the markers in *Xenopus* animal cap cells (Fig. 2C–2H).

**Figure 3:**
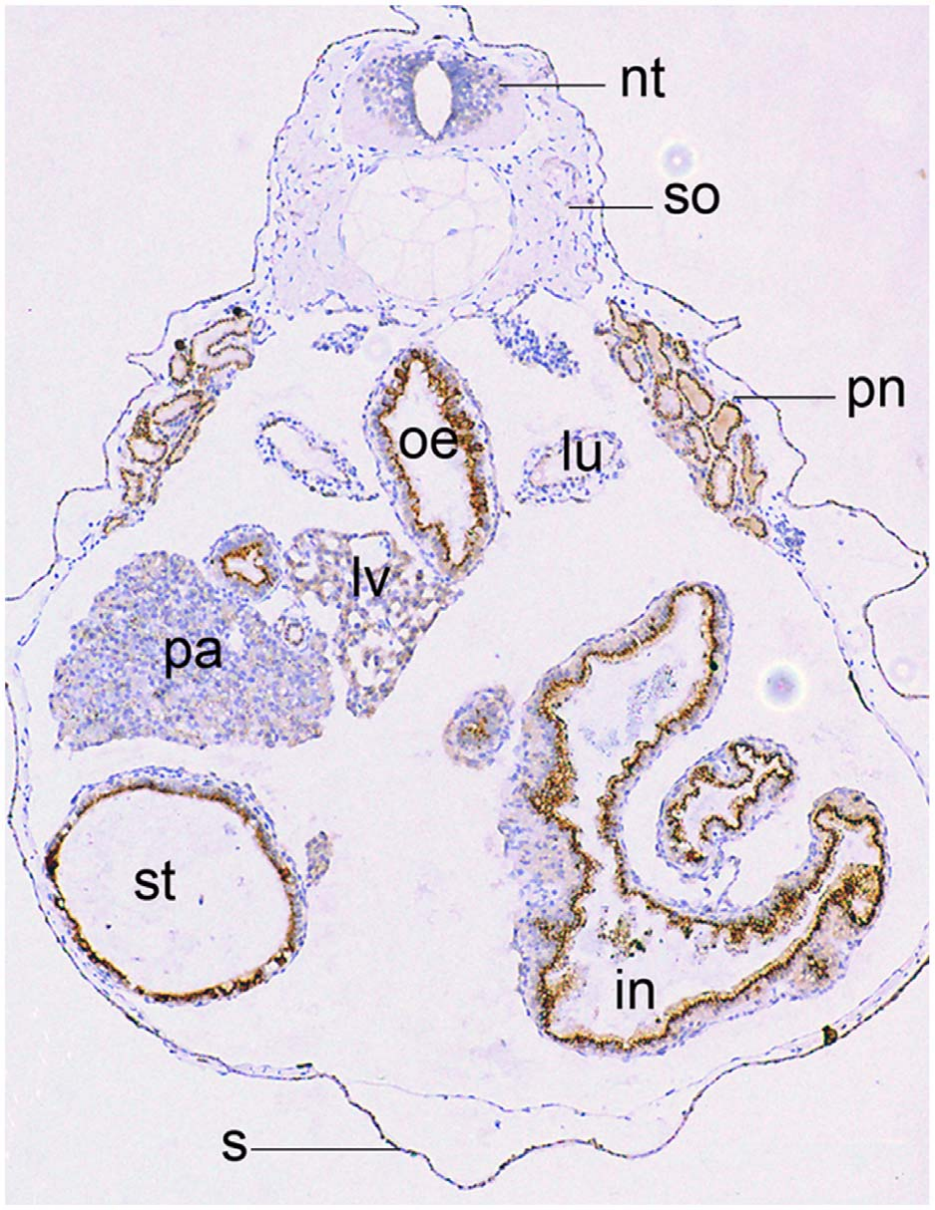
*Xenopus* golph2 is expressed in epithelial cells. Stage 46/47 embryos were fixed, embedded in paraffin, sectioned to a thickness of 3 µm. Endogenous golph2 was labeled with the primary antibody 10F12, followed by anti-mouse HRP-conjugated secondary antibody and stained with DAB solution; hematoxylin nuclear counterstaining. nt, neural tube; so, somites; pn, pronephros; oe, esophagus; lu, lung; lv, liver; pa, pancreas; st, stomach; i, intestine; s, skin.

Human GOLPH2 is expressed in epithelial cells [1]. To determine whether golph2 displays a similar cell type-specific expression, an immunocytochemistry analysis was performed on cross sections of stage 46/47 embryos. As shown in Fig. 3, high levels of golph2 were observed in the epithelial cells of the pronephric tubules, esophagus, stomach, intestine and skin.

These results showed that golph2 localizes to the Golgi, is highly expressed in epithelial cells and can form a complex with GOLPH2. Together, these results suggest that in addition to the similarity in amino acid sequence and predicted secondary structure of golph2 and GOLPH2, these two proteins may also have similar biological functions.

### 2. *Xenopus Golph2* is Expressed in the Pronephros

**Figure 4:**
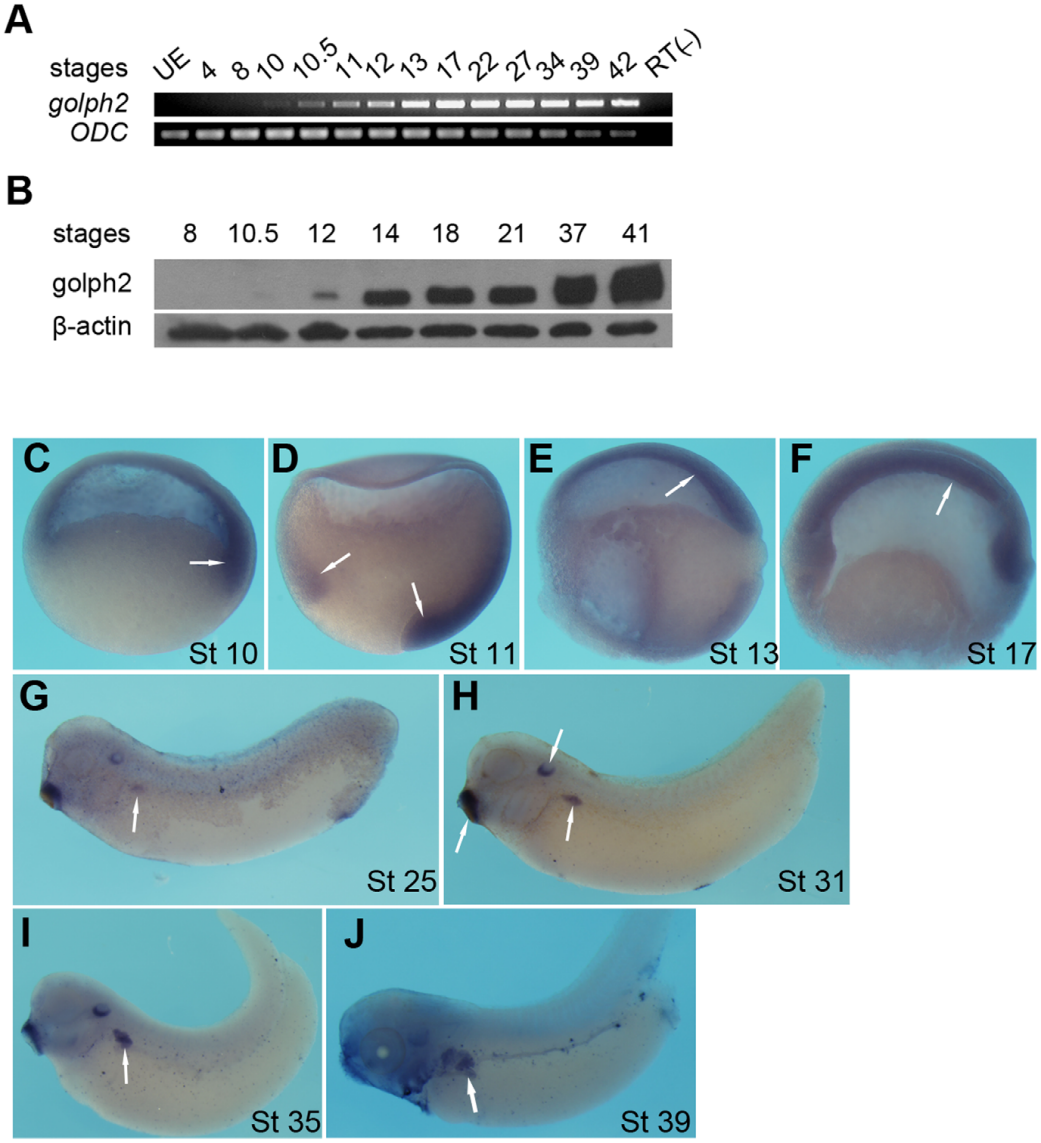
The expression pattern of *golph2* during early development. A. RT-PCR analysis of the temporal expression pattern of *golph2*. UE, unfertilized egg. RT(-), negative control of reverse transcription. *Ornithine decarboxylase* (*ODC*) was used as the RNA loading control. **B.** Western blotting analysis of golph2 expression during *Xenopus* embryogenesis. Beta-actin served as a protein loading control. **C–J.** Whole-mount *in situ* hybridization was conducted using a digoxigenin-labeled antisense probe for *golph2*. **C–F**. Embryos bisected prior to *in situ* hybridization. Orientation: animal top, dorsal right (C, D); dorsal top, ventral bottom (E, F), stages as indicated. The expression of *golph2* is detected in the dorsal marginal zone and the derivative mesoderm at the gastrula stage. **G–J**. Lateral view, anterior towards the left; *golph2* expression in the pronephros is detected between stages 25 and 39. Expression of *golph2* in the otic vesicle and cement gland is also shown (H). Arrow heads indicate the signal.

The temporal expression profile of *golph2* mRNA and protein during *Xenopus* early embryogenesis was analyzed by RT-PCR and Western blotting, respectively (Fig. 4A and 4B). The *golph2* transcripts were first weakly detected at the onset of gastrualiton, increased during gastrulation, and reached to a stable level at neurula and tailbud stages analyzed (Fig. 4A). In contrast, the first signal of golph2 protein was detected at midgastrulae and the expression level kept increasing during late stages analyzed (Fig. 4B).

To determine the spatial expression pattern of *golph2* during *Xenopus* development, whole-mount *in situ* hybridization was performed. As shown in Fig. 4, *golph2* transcripts were detected in the dorsal marginal zone during gastrulation (Fig. 4C–4E). At the early tailbud stage, when the pronephros anlagen form, *golph2* expression is observed in the pronephric region (Fig. 4G–4H). The expression pattern of *golph2* correlated with the stage at which the pronephros morphologically differentiates into the pronephric glomerulus and tubules [30]. At stages 31–35 (Fig. 4H–4I) the expression of *golph2* became restricted to the anterior pronephros. At the later tailbud stages (Fig. 4I), *golph2* expression was predominantly observed in the proximal tubules, with weaker expression observed in the distal tubules and duct. In addition to the pronephric region, *golph2* expression is also detected in the otic vesicle and cement gland (Fig. 4H).

In summary, during early *Xenopus* development, *golph2* is mainly expressed in the pronephros.

### 3. The Disruption of Golph2 Expression Results in Edema Formation

**Figure 5:**
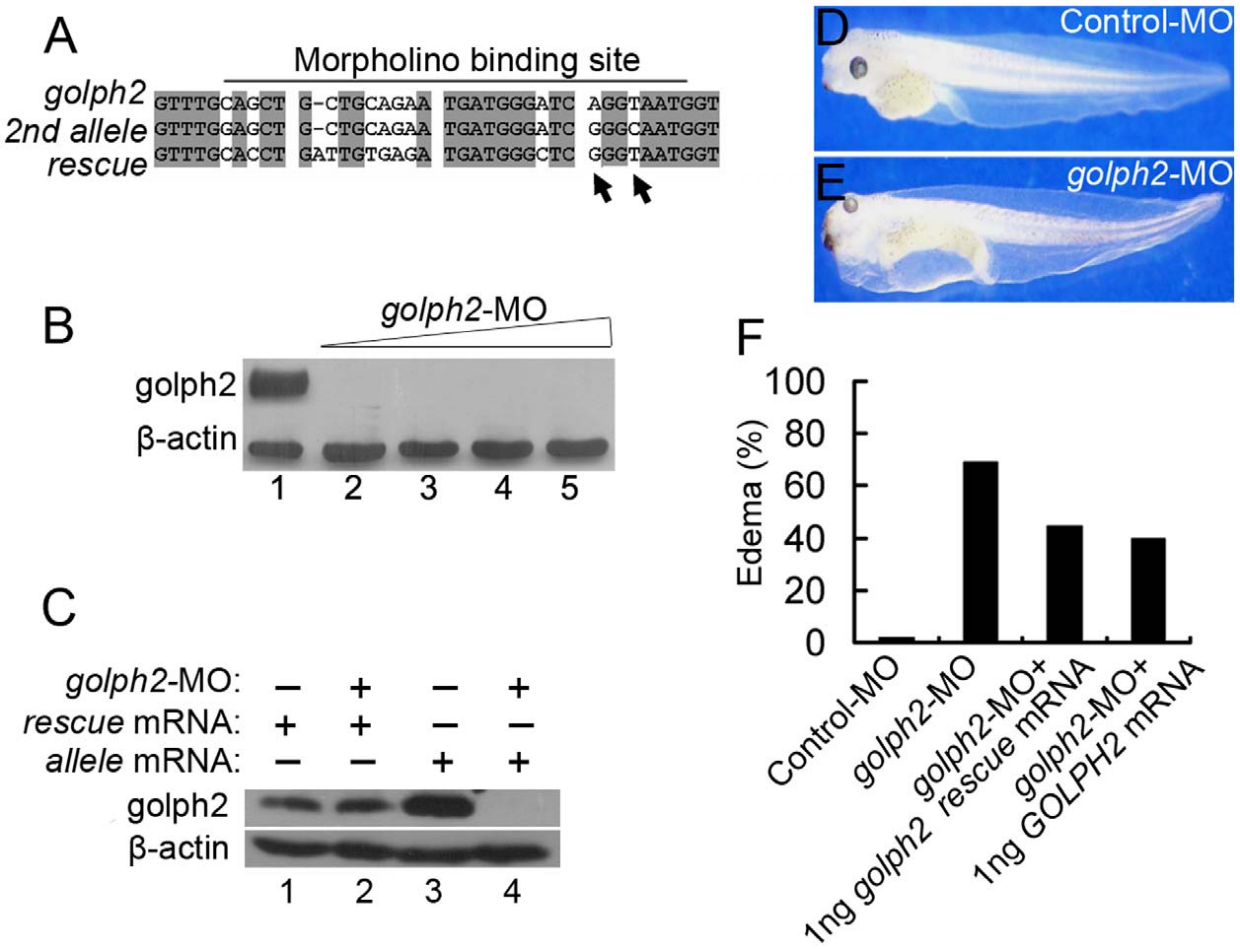
The inhibition of golph2 expression causes edema formation. A. Sequences of the two *golph2* pseudoalleles and the mRNA used for the rescue experiment. The arrow heads indicates the mismatch in the *golph2*-MO-targeting site of the second *golph2* allele. **B.** The expression of endogenous golph2 was inhibited by injection with morpholino into the vegetal pole of fertilized eggs. Western blotting analysis was performed at stage 33. Line 1∶56 ng of Control-MO; line 2∶16 ng of *golph2*-MO; line 3∶32 ng of *golph2*-MO; line 4∶48 ng of *golph2*-MO; line 5∶56 ng of *golph2*-MO. **C.** The specificity of *golph2*-MO was examined; 32 ng of *golph2*-MO was injected along with either 0.5 ng of *rescue* mRNA (line 2) or 0.5 ng of *allele* mRNA (line 4). *Xenopus* golph2 expression was detected in embryos of stage 10 using the antibody 10F12. **D.** Morphology of Control-MO-injected embryos. **E.** Morphology of *golph2*-MO-injected embryos displaying edema at the tadpole stage. **F.** Quantification of edema formation in embryos injected either with Control-MO (56 ng), *golph2*-MO (56 ng), *golph2*-MO (56 ng) with *golph2 rescue* mRNA (1 ng) or *golph2*-MO (56 ng) with human *GOLPH2* mRNA (1 ng).

To examine the role of golph2 during *Xenopus* development, a morpholino was designed to specifically block golph2 translation (Fig. 5A). To test its efficiency, 16 ng to 56 ng of *golph2*-MO was injected into the vegetal pole of the fertilized eggs. As shown in Fig. 5B, *golph2*-MO inhibited endogenous expression of golph2 to an undetectable level, even at lower dosages, relative to the Control-MO. Because *Xenopus laevis* is a pseudotetraploid, a gene may exist as two alleles. A second *golph2* allele (NCBI accession number BP696966) that contained two nucleotide mutations in the *golph2*-MO-targeting site was identified (Fig. 5A, indicated by an arrowheads), and *golph2*-MO could also efficiently target this second allele (Fig. 5C, compare lanes 3 and 4) but could not target the *rescue* mRNA (Fig. 5C, compare lanes 1 and 2).

To assess the loss of golph2 function during early *Xenopus* development, *golph2*-MO was injected into the vegetal pole of the fertilized eggs. The embryos form edema at later stages. The edema phenotype was mostly seen in embryos injected with higher dosages. Therefore, for displaying the edema phenotype, 56 ng of *golph2*-MO or Control-MO was injected. At stage 43, 69% of the *golph2*-MO-injected embryos (n = 128) developed edema (Fig. 5E) compared to only 2% of the Control-MO-injected embryos (n = 96) (Fig. 5D). To determine the specificity of this phenotype, 56 ng of *golph2*-MO was injected along with either 1 ng of *golph2 rescue* mRNA or 1 ng of human *GOLPH2* mRNA. Both mRNAs reduced the frequency of edema; 45% of the *golph2 rescue* mRNA-treated embryos (n = 112) and 40% of the *GOLPH2* mRNA-treated embryos (n = 123) developed edema (Fig. 5F).

### 4. *Xenopus* Golph2 is Required for the Terminal Differentiation of the Pronephros

**Figure 6:**
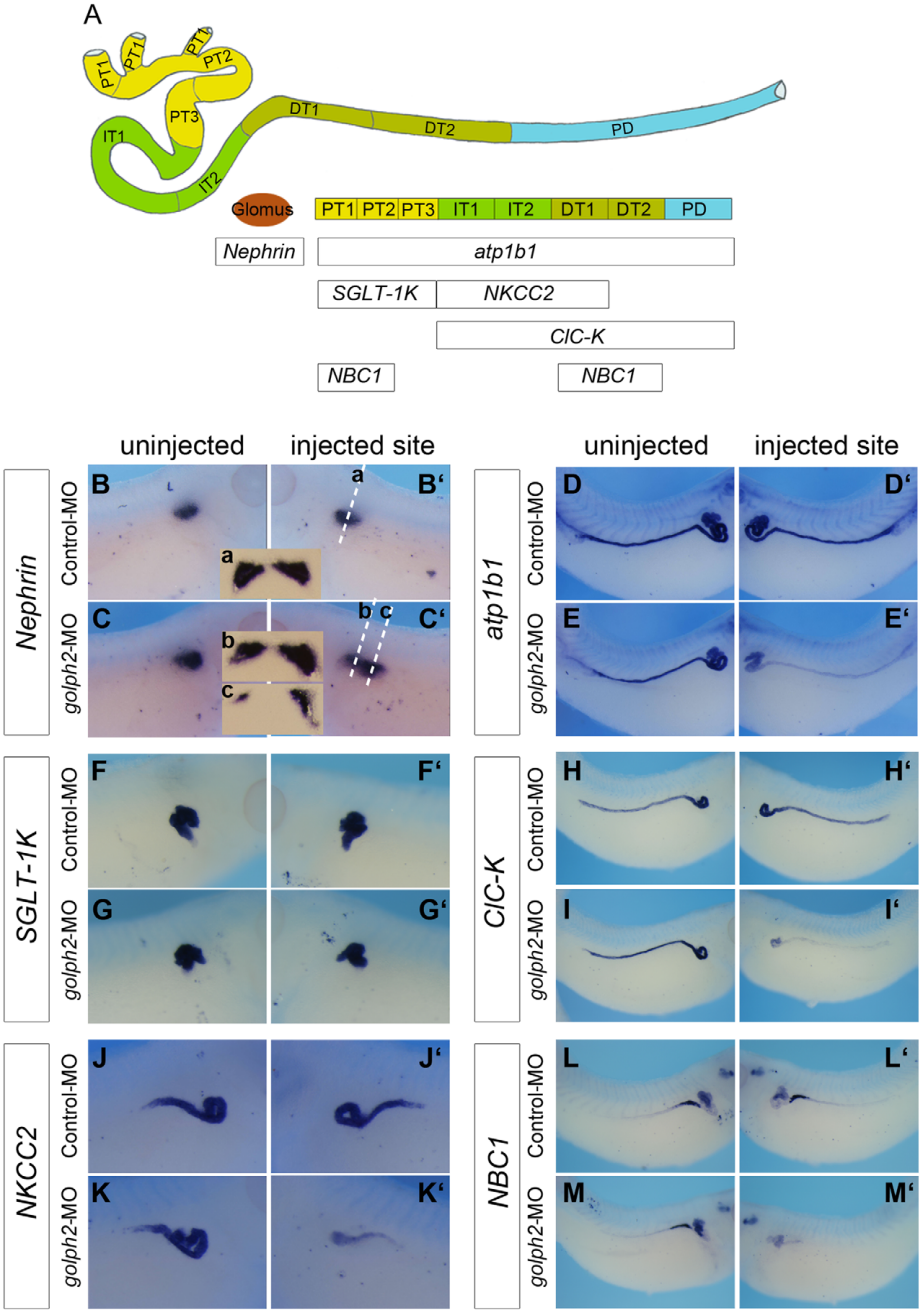
*Xenopus* golph2 is required for pronephros development. A. Schematic diagram of the pronephros with a summary of the pronephric marker genes that were used (lateral view, anterior towards the left). At stage 38, the pronephros is subdivided into distinct segments: the glomus, proximal tubule (PT1, PT2, PT3), intermediate tubule (IT1, IT2), distal tubule (DT1, DT2) and pronephric duct (PD). **B–M’.** Expression of the marker genes; the orientations of panels B to M show a lateral view, the anterior towards the right; the orientations of panels B’ to M’ show a lateral view, the anterior towards the left. A total of 32 ng of Control or *golph2* morpholino was injected into a single ventral blastomere of 4-cell-stage embryos from the lateral region; whole-mount *in situ* hybridization analysis was performed on stage 38. (B–C’) *Nephrin*; (a–c) transverse section of the embryos in panels B’ and C’; (D–E’) *atp1b1*; (F–G’) *SGLT-1K*; (H–I’) *ClC-K;* (J–K’) *NKCC2*; (L–M’) *NBC*1.

Defects in pronephros development have been reported to cause edema in *Xenopus* [18], [31]. To assess whether the inhibition of golph2 translation affects the pronephros development, we analyzed the expression of a panel of segment-specific marker genes (listed in Fig. 6A) along distinct regions of the pronephros. As shown in Fig. 6, 32 ng of *golph2*-MO, along with fluorescein-dextran as a lineage tracer, was targeted to the presumptive pronephric region by injection into one ventral blastomere of 4-cell-stage embryos from the lateral region [32]. Whole-mount *in situ* hybridization was performed using probes against the marker genes, and their expression in an uninjected site was compared with that of the *golph2*-MO-injected site in stage 38 embryos. The expression domain of *Nephrin*, a slit diaphragm-associated protein in the glomus, was increased in 61% of the embryos (n = 69; compare Fig. 6C to 6C’). In contrast, the expression of *atp1b1,* the beta-1 subunit of *Na+, K+-ATPase* present in the pronephric tubules and duct, decreased in 67% of the embryos (n = 58; compare Fig. 6E to 6E’). For *SGLT-1K*, a sodium glucose transporter present in the proximal tubule (Fig. 6A, PT1, PT2, and PT3), no difference in expression was observed (compare Fig. 6G to 6G’). However, expression of *ClC-K*, a chloride channel, in the intermediate-distal tubule and pronephric duct (Fig. 6A, IT1, IT2, DT1, DT2, and PD) decreased in 78% of the embryos (n = 108; compare Fig. 6I to 6I’). *NKCC2* is a sodium potassium co-transporter that is expressed in the intermediate and early distal tubule (Fig. 6A, IT1, IT2, and DT1). *NBC1* is a sodium bicarbonate transporter that is expressed in the early proximal and distal tubules of the pronephros (Fig. 6A, PT1, PT2, PT3, DT1, and DT2). At the *golph2*-MO injection site, the expression of *NKCC2* was reduced in 60% of the embryos (n = 74; compare Fig. 6K to 6K’), whereas *NBC1* expression was decreased in the distal tubule in 67% of the embryos (n = 40; compare Fig. 6M to 6M’).

**Figure 7:**
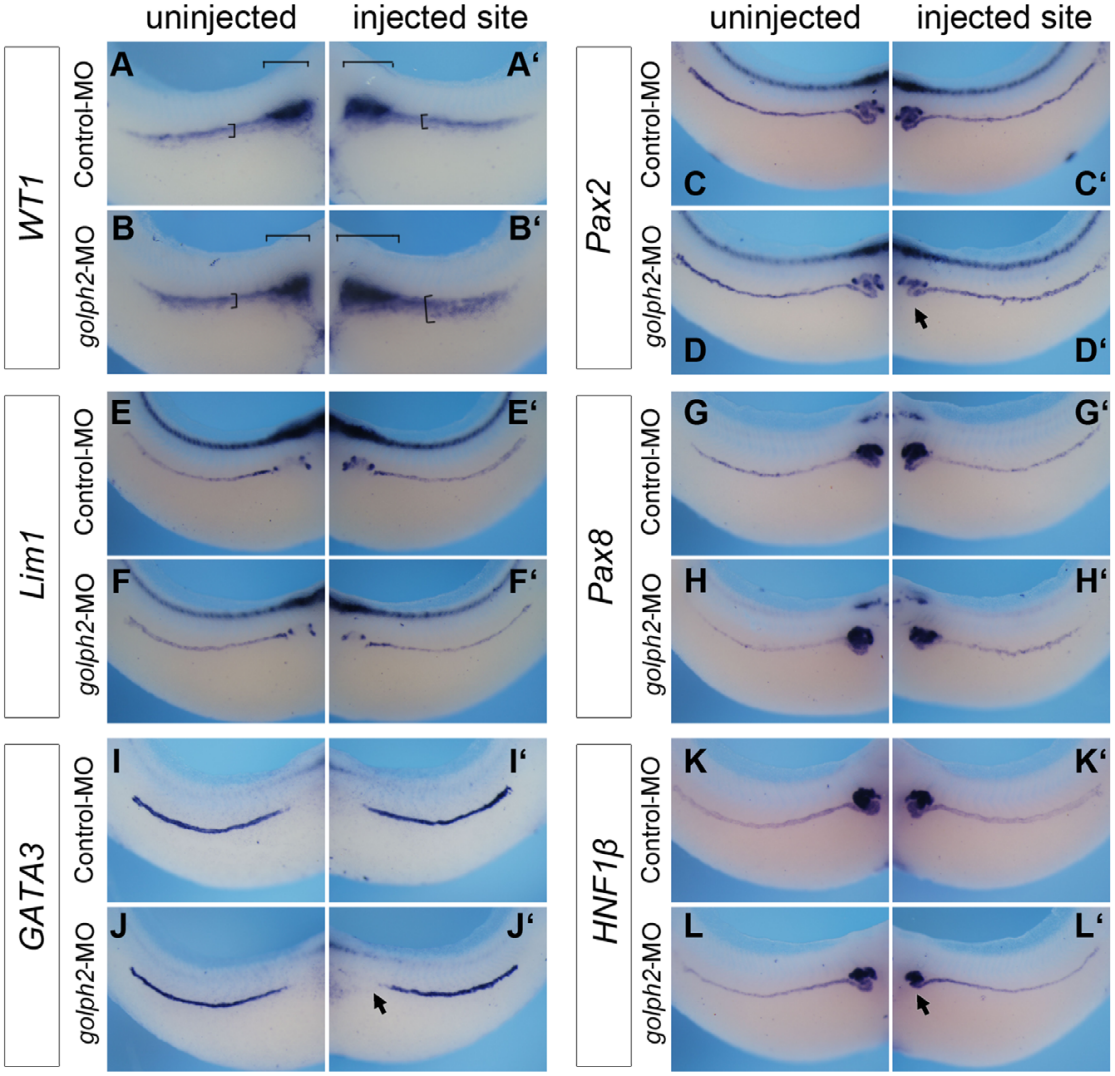
Expression analysis of transcription factors. A–L’, Whole-mount *in situ* hybridization analysis of Control and *golph2*-MO-injected embryos at stage 35; orientation: panels A–L, lateral view, the anterior towards the right; panels A’–L’, lateral view, the anterior towards the left. Morpholino (32 ng) was injected into a single ventral blastomere of 4-cell-stage embryos from the lateral region. (A–B’) *WT1*. Brackets indicate the expression domains. (C–D’), *Pax2*; (E–F’), *Lim1*; (G–H’), *Pax8*; (I–J’) *GATA3*; (K–L’), *HNF1β.* The arrow heads indicate reduced convolution of the pronephric tubules.

To determine whether the effect of *golph2*-MO is mediated by the altered expression of related transcription factors, the expression levels of *WT1, Pax2, Lim1, Pax8, GATA3* and *HNF1β* were analyzed by *in situ* hybridization at stage 35. As shown in Fig. 7A–7B’, the injection of *golph2*-MO resulted in the ectopic expression of *WT1* in both the glomus and the pronephric region in 52% of the embryos (n = 42; compare Fig. 7B to 7B’). Among other transcription factors tested, *HNF1β, GATA3,* and *Pax2* expression showed slight reduction on the injected side (Fig. 7L, 7L’; 7J, 7J’; and 7D, 7D’).

Together, these results suggest that deletion of golph2 altered the expression of both *WT1* and terminal differentiation genes in the pronephric tubules, duct and glomus.

### 5. Human GOLPH2 Rescues Defects Caused by *Golph2*-MO

**Figure 8:**
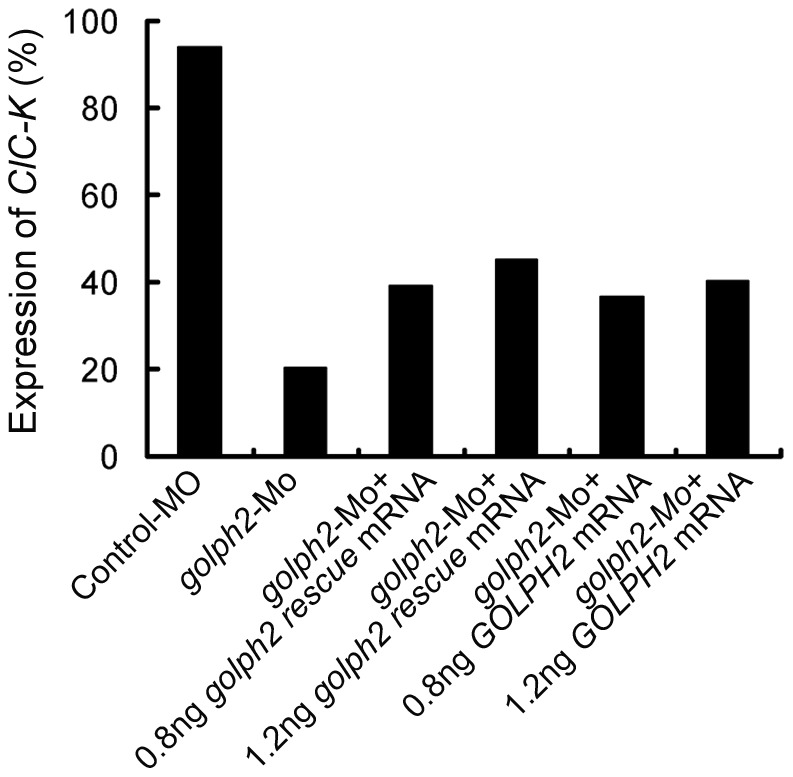
Both human and *Xenopus* golph2 can rescue the expression of the marker gene *ClC-K*. The percentage of embryos with normal levels of *ClC-K* expression relative to the total number of analyzed embryos (n). Control-MO (32 ng; n = 76); *golph2*-MO (32 ng; n = 86); *golph2*-MO (32 ng) with 0.8 ng of *golph*2 *rescue* mRNA (n = 33); *golph2*-MO (32 ng) with 1.2 ng of *golph2 rescue* mRNA (n = 66); *golph2*-MO (32 ng) with 0.8 ng of *GOLPH2* mRNA (n = 33); *golph2*-MO (32 ng) with 1.2 ng of *GOLPH2* mRNA (n = 55).

Previous results indicated that injection of *GOLPH2* mRNA could partially reverse edema induced by *golph2*-MO (Fig. 5F), thereby suggesting that GOLPH2 could partially complement golph2 function. To further confirm this observation, *GOLPH2* mRNA was co-injected with *golph2*-MO in one ventral blastomere of 4-cell-stage embryos, and the percentage of embryos with normal *ClC-K* expression was measured by whole-mount *in situ* hybridization at stage 39. As shown in Fig. 8, 94% of the Control-MO-injected embryos (n = 76) expressed *ClC-K* normally on the injected site compared to 20% of the *golph2*-MO-injected embryos (n = 86). Both *golph2 rescue* mRNA and *GOLPH2* mRNA rescued the *CIC-K* expression in the pronephric tubules and pronephric duct. The percentages are (n = total injected embryos): 0.8 ng of *golph2 rescue* mRNA, 40% (n = 33); 1.2 ng of *golph2 rescue* mRNA, 45% (n = 66); 0.8 ng of *GOLPH2* mRNA, 37% (n = 33); 1.2 ng of *GOLPH2* mRNA, 41% (n = 55). This result indicates that both golph2 and GOLPH2 could partially rescue the phenotype observed when endogenous golph2 expression is blocked.

## Discussion

It has been shown that GOLPH2 is upregulated in a number of tumors and is an emerging biomarker for liver diseases [33]. Therefore, it is important to understand the biological function of GOLPH2 and its role in pathogenesis. Based on the high level of sequence conservation among vertebrates, it has been suggested that the biological function of GOLPH2 is also conserved. Therefore, it is reasonable to study the biological function of GOLPH2 using model organisms, such as *Xenopus laevis*.

### *Xenopus* as a Model System for the Study of GOLPH2 Function

In this report, we provided experimental evidence confirming that golph2 shares characteristic properties with human GOLPH2. We showed that golph2 exists as a dimer that is capable of associating with GOLPH2 to form a hetero-complex, thereby suggesting that the two proteins are structurally similar. The coiled-coil domain, which is one of the most conserved regions of GOLPH2, has been shown to be the determinant for both dimer formation and interactions with other protein (secretory clusterin) [34]. Thus, it is reasonable to predict that golph2 interaction pathways (i.e., networks) identified in *Xenopus* might also be shared by GOLPH2 in humans.

Other conserved regions of GOLPH2 include the TMD and positively charged residues on the cytoplasmic side. These structural features are the determinants for the intracellular localization and transportation of GOLPH2. We demonstrated that endogenous golph2 localizes to Golgi in *Xenopus* cells. It is hypothesized that golph2 could also be trafficked between the Golgi and plasma membrane through an endosomal pathway, cleaved by proprotein protease and secreted when expression is up-regulated, as found in studies of GOLPH2 [35]. The epithelial-specific expression of golph2 is interesting. This finding suggests that in addition to similarity in sequences and protein structures, the expression of GOLPH2 and golph2 are regulated by similar mechanisms.

In summary, the results presented in this report support the notion that *Xenopus* model could be use to study the function of GOLPH2.

### *Xenopus* Golph2 is Required for the Pronephros Development

In this report, we showed that golph2 plays a critical role in the terminal differentiation of the *Xenopus* pronephros. The elimination of golph2 using translation-specific morpholino oligomers resulted in the expansion of glomus size and reduced the expression of transport membrane proteins along the pronephric tubules and duct.

During the early development of *Xenopus*, we demonstrated that *golph2* is expressed after the mid-blastula transition, in the dorsal blastopore lip and the derivatives from mesoderm (notochord and head endomesoderm) at the gastrula stage. The Spemann Organizer region secretes a cocktail of BMP antagonists, and the GOLPH2 protein has been reported to be secreted [35], [36]. However, protein sequence predictions have not indicated the presence of any cysteine residues or CR motifs, which are common in the BMP antagonists [37], and the injection of *golph2* mRNA into embryos does not induce the expansion of the dorsal organ (data not shown). Additionally, *golph2* transcripts were detected in the cement gland, which is a mucus-secreting organ. It has been presumed that GOLPH2 serves as a Golgi resident protein that may be involved in the trafficking or modification of secreted proteins. In the tailbud stages, *golph2* was expressed in the pronephros during proximal-distal differentiation and tubule extension. This distinct expression pattern suggested a spatiotemporal requirement of *golph2* in pronephros development.

In the *Xenopus* model, the experimental block of golph2 translation by specific antisense morpholino oligomers caused edema in tadpole-stage embryos. Members of the *Xenopus* have adapted to the constant influx of water from the skin and gut by excreting large volumes of urine. The impaired pronephros may result in water retention and an edema phenotype [31]. Both *golph2* and *GOLPH2* mRNA could partially rescue the edema formation. To obtain a more detailed understanding, we analyzed a series of marker genes for the proximal-distal patterning of pronephros. The depletion of golph2 results in an expanded size of the glomus, reduced tubular convolution and decreased expression of transport proteins along the pronephric tubules and duct. These results implicated golph2 in pronephros patterning and terminal differentiation of the tubules. It has been shown that several transcription factors are expressed in response to nephrogenesis. Among the factors that we examined, *WT1* is expressed around the anterior border of the pronephros [25] and is regarded as the key factor in the regulation of glomus development by the suppression of tubule and duct gene expression [38], [39], [40]. In the *golph2*-MO-injected site of the embryos, *WT1* expression was increased in the glomus and laterally expanded. Together with the observed changes in marker gene expression, these results suggest that ectopic *WT1* induces the expression of *Nephrin* and suppresses the expression of pronephros transporters in the tubules. It has been reported that *WT1* represses the expression of *Pax2* during kidney development [41]. The ectopic *WT1* in the lateral region may inhibit the expression of the other transcript factors in the pronephric tubules. Using a mouse model, a previous study provided evidence for a role of GOLPH2 in kidney function. The transgenic mouse with truncated GOLPH2 developed a glomerular disease, i.e., focal segmental glomerulosclerosis (FSGS), based on the observation of histological changes [8]. FSGS is a broadly defined pattern of injury, whereas genetic defects are revealed in clinical studies. It has been shown that mutations in *Nephrin*, *ClC-K* and *NKCC2* are related to this disease [42], [43], [44].

It has been reported that Notch signaling was involved in the segmentation patterning of the pronephros along the proximal-distal axis. Studies in *Xenopus* indicated that the activation of Notch signaling promotes glomus formation but inhibits tubulogenesis [13]. In addition, ectopic expression of *WT1* is induced by Notch signaling, and the downstream Notch effector gene, hey1, is regulated by *WT1* [14]. This suggests that golph2 may mediate Notch signaling during pronephros development. However, the Notch-mediated lateral inhibition was not detected on the *golph2*-MO injected site (data not shown). On the other hand, Notch receptors are coated with a variety of glycans; the O-fucose glycans modulate the binding between Notch and its ligands [45], mutations that prevent the glycans synthesis cause signaling defects. The Golgi protein fringe, which transfers GlcNAC onto fucose in Notch EGF repeats, was demonstrated to mediate Notch signaling during *Drosophila* and vertebrate development [46]. Whether golph2 affects Notch signaling via modification of Notch proteins requires further investigation.

In conclusion, we propose the use of *Xenopus laevis* as a model system for the study of golph2 function. Our results suggest that golph2 is required for pronephros development. The utilization of a well-defined model system such as *Xenopus laevis* will lead to a better understanding of the biological functions, expression regulation mechanisms, pathogenicity and other important aspects of GOLPH2.

## Supporting Information

Figure S1 ***Xenopus*** **golph2 is conserved with other vertebrate GOLPH2 sequences.** The alignment of golph2 with human and mouse GOLPH2 sequences using ClustalW showed high conservation in the N-terminus of the protein. The accession numbers are: *Homo sapiens*, NP_057632; *Mus musculus*, BAE39697; *Xenopus laevis*, JF79249.
(TIF)

Table S1 **Primer sequences for the required molecular markers.**
(DOC)

## References

[1] Kladney RD, Bulla GA, Guo LS, Mason AL, Tollefson AE (2000). GP73, a novel Golgi-localized protein upregulated by viral infection.. Gene.

[2] Kladney RD, Cui XY, Bulla GA, Brunt EM, Fimmel CJ (2002). Expression of GP73, a resident Golgi membrane protein, in viral and nonviral liver disease.. Hepatology.

[3] Iftikhar R, Kladney RD, Havlioglu N, Schmitt-Graff A, Gusmirovic I (2004). Disease- and cell-specific expression of GP73 in human liver disease.. Am J Gastroenterol.

[4] Fritzsche FR, Riener MO, Dietel M, Moch H, Jung K (2008). GOLPH2 expression in renal cell cancer.. BMC Urol.

[5] Wei S, Dunn TA, Isaacs WB, De Marzo AM, Luo J (2008). GOLPH2 and MYO6: putative prostate cancer markers localized to the Golgi apparatus.. Prostate.

[6] Gong Y, Long Q, Xie H, Zhang T, Peng T (2012). Cloning and characterization of human Golgi phosphoprotein 2 gene (GOLPH2/GP73/GOLM1) promoter.. Biochem Biophys Res Commun.

[7] Hu L, Li L, Xie H, Gu Y, Peng T (2011). The Golgi localization of GOLPH2 (GP73/GOLM1) is determined by the transmembrane and cytoplamic sequences.. PLoS One.

[8] Wright LM, Yong S, Picken MM, Rockey D, Fimmel CJ (2009). Decreased survival and hepato-renal pathology in mice with C-terminally truncated GP73 (GOLPH2).. Int J Clin Exp Pathol.

[9] Saxén L (1987). Organogenesis of the Kidney.. Cambridge, UK: Cambridge University Press.

[10] Vize PD, Woolf A, Bard J (2003). The Kidney: From Normal Development to Congenital Diseases.. Amsterdam: Academic Press.

[11] Zhou X, Vize PD (2004). Proximo-distal specialization of epithelial transport processes within the Xenopus pronephric kidney tubules.. Dev Biol.

[12] Reggiani L, Raciti D, Airik R, Kispert A, Brandli AW (2007). The prepattern transcription factor Irx3 directs nephron segment identity.. Genes Dev.

[13] McLaughlin KA, Rones MS, Mercola M (2000). Notch regulates cell fate in the developing pronephros.. Dev Biol.

[14] Taelman V, Van Campenhout C, Solter M, Pieler T, Bellefroid EJ (2006). The Notch-effector HRT1 gene plays a role in glomerular development and patterning of the Xenopus pronephros anlagen.. Development.

[15] Wessely O, Tran U (2011). Xenopus pronephros development-past, present, and future.. Pediatric Nephrology.

[16] Dressler GR (2006). The cellular basis of kidney development.. Annu Rev Cell Dev Biol.

[17] Jones EA (2005). Xenopus: a prince among models for pronephric kidney development.. J Am Soc Nephrol.

[18] Tran U, Pickney LM, Ozpolat BD, Wessely O (2007). Xenopus Bicaudal-C is required for the differentiation of the amphibian pronephros.. Dev Biol.

[19] Le Bot N, Antony C, White J, Karsenti E, Vernos I (1998). Role of xklp3, a subunit of the Xenopus kinesin II heterotrimeric complex, in membrane transport between the endoplasmic reticulum and the Golgi apparatus.. J Cell Biol.

[20] Zhang F, Gu Y, Li X, Wang W, He J (2010). Up-regulated Golgi phosphoprotein 2 (GOLPH2) expression in lung adenocarcinoma tissue.. Clin Biochem.

[21] Wen L, Yang Y, Wang Y, Xu A, Wu D (2010). Appl1 is essential for the survival of Xenopus pancreas, duodenum, and stomach progenitor cells.. Dev Dyn.

[22] Nieuwkoop P, Faber J (1994). Normal table of *Xenopus laevis*.. New York: Garland Publishing, Inc.

[23] Gerth VE, Zhou X, Vize PD (2005). Nephrin expression and three-dimensional morphogenesis of the Xenopus pronephric glomus.. Dev Dyn.

[24] Vize PD (2003). The chloride conductance channel ClC-K is a specific marker for the Xenopus pronephric distal tubule and duct.. Gene Expr Patterns.

[25] Carroll TJ, Vize PD (1996). Wilms’ tumor suppressor gene is involved in the development of disparate kidney forms: evidence from expression in the Xenopus pronephros.. Dev Dyn.

[26] Carroll TJ, Vize PD (1999). Synergism between Pax-8 and lim-1 in embryonic kidney development.. Dev Biol.

[27] Wild W, Pogge von Strandmann E, Nastos A, Senkel S, Lingott-Frieg A (2000). The mutated human gene encoding hepatocyte nuclear factor 1beta inhibits kidney formation in developing Xenopus embryos.. Proc Natl Acad Sci U S A.

[28] Harland RM (1991). In situ hybridization: an improved whole-mount method for Xenopus embryos.. Methods Cell Biol.

[29] Li XJ, Han DD, Kam RKT, Guo XG, Chen M (2010). Developmental Expression of sideroflexin Family Genes in Xenopus Embryos.. Developmental Dynamics.

[30] Vize PD, Seufert DW, Carroll TJ, Wallingford JB (1997). Model systems for the study of kidney development: Use of the pronephros in the analysis of organ induction and patterning.. Developmental Biology.

[31] Howland RB (1916). On the Effect of Removal of the Pronephros of the Amphibian Embryo.. Proc Natl Acad Sci U S A.

[32] Tena JJ, Neto A, de la Calle-Mustienes E, Bras-Pereira C, Casares F (2007). Odd-skipped genes encode repressors that control kidney development.. Dev Biol.

[33] Gu Y, Chen W, Zhao Y, Chen L, Peng T (2009). Quantitative analysis of elevated serum Golgi protein-73 expression in patients with liver diseases.. Ann Clin Biochem.

[34] Zhou Y, Li LK, Hu LB, Peng T (2011). Golgi phosphoprotein 2 (GOLPH2/GP73/GOLM1) interacts with secretory clusterin.. Molecular Biology Reports.

[35] Bachert C, Fimmel C, Linstedt AD (2007). Endosomal trafficking and proprotein convertase cleavage of cis Golgi protein GP73 produces marker for hepatocellular carcinoma.. Traffic.

[36] Romano PR, Nikolaeva OV, Steel L, Fimmel CJ, Marrero J (2003). GP73, a resident Golgi membrane protein, appears in sera of patients with viral liver disease and hepatocellular cancer.. Hepatology.

[37] De Robertis EM, Larrain J, Oelgeschlager M, Wessely O (2000). The establishment of Spemann’s organizer and patterning of the vertebrate embryo.. Nature Reviews Genetics.

[38] Kreidberg JA, Sariola H, Loring JM, Maeda M, Pelletier J (1993). Wt-1 Is Required for Early Kidney Development.. Cell.

[39] Van Campenhout C, Nichane M, Antoniou A, Pendeville H, Bronchain OJ (2006). Evi1 is specifically expressed in the distal tubule and duct of the Xenopus pronephros and plays a role in its formation.. Dev Biol.

[40] Perner B, Englert C, Bollig F (2007). The Wilms tumor genes wt1a and wt1b control different steps during formation of the zebrafish pronephros.. Dev Biol.

[41] Ryan G, Steele-Perkins V, Morris JF, Rauscher FJ, 3rd, Dressler GR (1995). Repression of Pax-2 by WT1 during normal kidney development.. Development.

[42] Chiang CK, Inagi R (2010). Glomerular diseases: genetic causes and future therapeutics.. Nat Rev Nephrol.

[43] Bettinelli A, Borsa N, Syren ML, Mattiello C, Coviello D (2005). Simultaneous mutations in the CLCNKB and SLC12A3 genes in two siblings with phenotypic heterogeneity in classic Bartter syndrome.. Pediatr Res.

[44] Yamazaki H, Nozu K, Narita I, Nagata M, Nozu Y (2009). Atypical phenotype of type I Bartter syndrome accompanied by focal segmental glomerulosclerosis.. Pediatric Nephrology.

[45] Moloney DJ, Panin VM, Johnston SH, Chen J, Shao L (2000). Fringe is a glycosyltransferase that modifies Notch.. Nature.

[46] Stanley P, Okajima T (2010). Roles of Glycosylation in Notch Signaling.. Notch Signaling.

