# Supplementary figures and images for: Xenopus as a Model System for the Study of GOLPH2/GP73 Function: Xenopus golph2 Is Required for Pronephros Development
Source: PLoS One. 2012 Jun 14;7(6):e38939. doi: 10.1371/journal.pone.0038939 (PMC3375297; doi:10.1371/journal.pone.0038939)

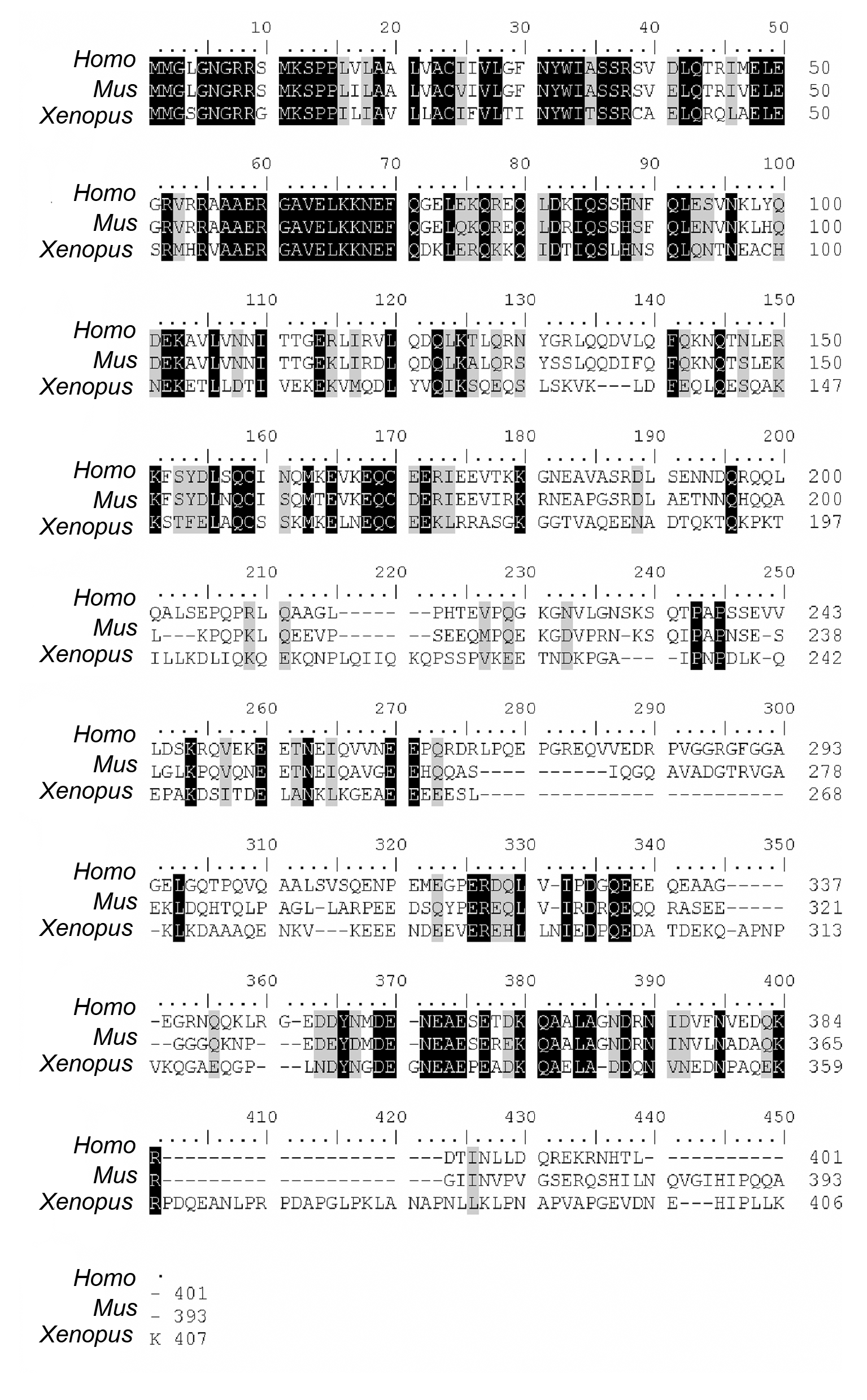

Supplement: Figure S1 — Xenopus golph2 is conserved with other vertebrate GOLPH2 sequences. The alignment of golph2 with human and mouse GOLPH2 sequences using ClustalW showed high conservation in the N-terminus of the protein. The accession numbers are: Homo sapiens, NP_057632; Mus musculus, BAE39697; Xenopus laevis, JF79249. (TIF) [file pone.0038939.s001.tif]
