# Supplementary material for: Xenopus as a Model System for the Study of GOLPH2/GP73 Function: Xenopus golph2 Is Required for Pronephros Development
Source: PLoS One. 2012 Jun 14;7(6):e38939. doi: 10.1371/journal.pone.0038939 (PMC3375297; doi:10.1371/journal.pone.0038939)
Supplement: Table S1 — Primer sequences for the required molecular markers. (DOC) [file pone.0038939.s002.doc]

**Table S1. Primer sequences for the required molecular markers**

| **Gene** | **GenBank Accession number** | **Forward primer (5’-3’)** | **Reverse primer (5’-3’)** |
| --- | --- | --- | --- |
| *Nephrin* | NM_001095706 | ACGTGAGAGTCGACGACTCTGGCATTTACA | GTAATCATCGCATGCTTCCGCATACTCGT |
| *atp1b1* | NM_001086759 | CCTTCGTCGACCTAAAGAAGCCACAACCCT | GGGTCGTACGCATGCTCAGCTACTCTTTATATCA |
| *SGLT-1K* | CA788193 | GGAATTCTTGCGGTCGACTAGCACAGAAACCCCTTCATAAGAGGAT | GTATATGGCATGCGGTGTTTGGAACAGTTATCTTTGGCATTGCT |
| *ClC-K* | NM_001085839 | GGATCGAGTCGACATGAGCCGTGT | AGGATGCATGCGATCTGTCCCGTA |
| *NKCC2* | JQ676997 | TGATGGTCGACATCACCTGGTGGCTGCTCT | TGAATAAAATGTTAGTACATTCTGTGA |
| *NBC1* | BU905206 | TGATGCTGTCGACAAGATGATCAAGTTTGCTGACTATTACCCAA | AAATGCATGCCAATGGGAGGATTGACCAGTTAGCCAG |
| *WT1* | NM_001085867 | AATCTGTCGACTCCAGTCTCTAGTCTGTCCGGGA | CTAAAGGGCATGCTGGAGTTTGGTCATGTTCCT |
| *Pax2* | AF027769 | TGAAGTTCAGTCCAGAGGCGAAACGGCGAC | CGTCAGTAGGGAAAGCAGAGTAGGCCACTC |
| *Lim1* | NM_001090659 | ATAACCAGTCGACATGGTTCACTGTGCTGGATGCGA | GTCCAGGGCATGCTACCACACTGCCGTTTCGTT |
| *Pax8* | NM_001088472 | GATGGTCGACAGCAGCATCAGATCAGAGCTGAC | ATAGGCGCATGCCGTTGTGGGAGGAGGT |
| *GATA3* | NM_001090866 | AGACAAGTGCGTATTGGTGCTGCTGGCC | GGTACAAAGACTAACCCATTGCAGTGACCATACTGGAT |
| *HNF1β* | NM_001089811 | GGACAGCCTGATGATACCTGC | TCACCATGCTTGCAAAGGAC |
